# Reading Comprehension in a Large Cohort of French First Graders from Low Socio-Economic Status Families: A 7-Month Longitudinal Study

**DOI:** 10.1371/journal.pone.0078608

**Published:** 2013-11-08

**Authors:** Edouard Gentaz, Liliane Sprenger-Charolles, Anne Theurel, Pascale Colé

**Affiliations:** 1 Laboratoire de Psychologie et NeuroCognition (Centre National de Recherche Scientifique), Université Pierre Mendès-France, Grenoble, France; 2 Faculté de Psychologie et Sciences de l’Education, Université de Genève, Genève, Suisse; 3 Laboratoire de Psychologie Cognitive (CNRS), Aix-Marseille Université, Marseille, France; 4 Laboratoire de Psychologie de la Perception (CNRS), Paris Descartes, Paris, France; National University of Singapore, Singapore

## Abstract

**Background:**

The literature suggests that a complex relationship exists between the three main skills involved in reading comprehension (decoding, listening comprehension and vocabulary) and that this relationship depends on at least three other factors orthographic transparency, children’s grade level and socioeconomic status (SES). This study investigated the relative contribution of the predictors of reading comprehension in a longitudinal design (from beginning to end of the first grade) in 394 French children from low SES families.

**Methodology/Principal findings:**

Reading comprehension was measured at the end of the first grade using two tasks one with short utterances and one with a medium length narrative text. Accuracy in listening comprehension and vocabulary, and fluency of decoding skills, were measured at the beginning and end of the first grade. Accuracy in decoding skills was measured only at the beginning. Regression analyses showed that listening comprehension and decoding skills (accuracy and fluency) always significantly predicted reading comprehension. The contribution of decoding was greater when reading comprehension was assessed via the task using short utterances. Between the two assessments, the contribution of vocabulary, and of decoding skills especially, increased, while that of listening comprehension remained unchanged.

**Conclusion/Significance:**

These results challenge the ‘simple view of reading’. They also have educational implications, since they show that it is possible to assess decoding and reading comprehension very early on in an orthography (i.e., French), which is less deep than the English one even in low SES children. These assessments, associated with those of listening comprehension and vocabulary, may allow early identification of children at risk for reading difficulty, and to set up early remedial training, which is the most effective, for them.

## Introduction

The goal of reading is the understanding of written texts, and the acquisition of this ability is crucial in our society in which the written word is omnipresent. Despite intensive instruction, many early-grade children fail to reach functional levels in reading comprehension, with a large number of these children coming from low socio-economic status (SES) families [1]–[4]. According to the simple view of reading [5], reading comprehension in typically developing children critically depends on the product of decoding and listening comprehension skills. Research in this area suggests that reading comprehension involves a complex relationship between these two skills. This relationship may also depend on three additional factors: the consistency of the relationship between the basic units of written and spoken language (i.e. between graphemes and phonemes in an alphabetic writing system [6]–[7]), children’s grade level, and families’ SES.

Despite widespread acceptance of the simple view of reading, some studies have reported results supporting a ‘not-so-simple view’ of the skills underlying reading comprehension: (a) reading comprehension and decoding are relatively independent [8]–[12]; (b) other skills, particularly vocabulary, are important for reading comprehension [13]–[19]; (c) the contribution of the predictors involved in reading comprehension, depends on children’s grade level: between early and later grades, the contribution of listening comprehension increased while that of decoding decreased [14] [19]–[20].

However, these results were mostly found in studies on English-speaking children, a language with an ‘outlier orthography’ [21]. The results obtained in languages whose orthography is not so deep (e.g. Dutch and French) suggest that the consistency of the orthography affects the weight of abilities that contribute to reading comprehension. For instance, in a longitudinal study on Dutch children [22], it was reported that decoding speed and listening comprehension measured in grade 1 had specific effects on reading comprehension measured in grade 3. Interestingly, in this study, as in another study on Dutch children [17], the effect on reading comprehension of listening comprehension tended to be greater than that of decoding in the first grades. In a French study, listening comprehension also appeared to be a more powerful predictor of reading comprehension than decoding as early as the end of first grade [12]. These results [12] [17] [22] contrast sharply with those reported in English [14] [20].

There are few studies including a measure of oral vocabulary in addition to a measure of listening comprehension [13]–[17]: they all involved English children, except one with Dutch children [17]. In two other studies, only oral vocabulary but not listening comprehension was assessed [18]–[19]. Two main results emerge from this work: the weight of vocabulary on reading comprehension depends on (1) the children’s grade level and (2) the consistency of the orthography. Indeed, the study on Dutch children [17] showed that vocabulary in grade 1 influenced reading comprehension in grade 2. Alternatively, some studies on English children showed no contribution of vocabulary in grade 1 [14] but a significant one in subsequent grades, for instance in grades 4 [15] [18], 6 [14], 7 and 9 [15]. However, early vocabulary level (assessed at the beginning of kindergarten) was found to predict early reading comprehension performances (assessed at the beginning of grade 2) in other studies with English children [13]. Inconsistent results were observed even among English children with poor skills in reading comprehension, the level of vocabulary distinguishing these children from control children in some studies [19] but not always in others [16]. These inconsistent results do not seem to be due to the specific test used to assess vocabulary: either receptive vocabulary assessed by the choice of the picture corresponding to a word said by the examiner [13]–[14] [17]–[18], which is the typical assessment of vocabulary according to some researchers [13], or productive vocabulary assessed with word definition [14]–[15] [18]–[19]. Indeed, receptive vocabulary was found to predict reading comprehension in some studies [13] [17]–[18], but not in other studies [14]. Similarly, productive vocabulary was found to predict reading comprehension in some studies [15] [18]–[19], but not in others [14]. Two biases may explain the higher weight on reading comprehension of vocabulary in some studies: the fact that listening comprehension was not taken into account [18]–[19] or the use of written words to assess vocabulary [17]–[18].

In addition, the predictive power of vocabulary depends on the means used to assess decoding. As explained by Muter and his colleagues [13] (p.667) *“Gough and Tunmer (1986), in their simple model of reading proposed that the ability to comprehend what was read depended on both word recognition ability (or in their terms, decoding) and language comprehension (assessed by a measure of listening comprehension)”*. As noted also by Ouellette and Beers [14] (p.203) *“the use of the term decoding within the simple view may be misleading”* since, in their study, both the reading of nonsense words (called here after pseudowords) and of high frequency irregular words explained reading comprehension. Decoding and visual word recognition should thus be distinguished. ‘Decoding’ should be defined as a procedure allowing the reader to assess written items by the use of grapheme-phoneme correspondences. ‘Written word recognition’ should be defined as a procedure allowing the reader to access the words that are in their lexicon. The first procedure is assessed by the reading of pseudowords, the second by the reading of high frequency words, usually, irregular words, because they cannot be decoded whereas high frequency regular words could be read either by decoding or by visual word recognition. Both oral vocabulary and listening comprehension were assessed together with decoding and/or visual word recognition in four studies [13]–[15] [17]. In the early grades [13]–[14] [17], the contribution of vocabulary was non-significant when decoding (pseudoword reading) and visual word recognition (high frequency irregular words reading) were separately integrated in the model [14]. That contribution was significant when only visual word recognition was integrated in the model but was weaker when the list of words included high frequency irregular words [13] than with a list with only high frequency regular words [17]. It is therefore important to assess pure decoding skills using pseudoword reading, especially in studies with young children. The non-significant contribution of vocabulary observed in Ouellette and Beers [14] could be due to the strong links between irregular word reading and reading comprehension.

Moreover, there is recent evidence showing that commonly used tests of reading comprehension do not all tap into the same cognitive processes. A study with English participants [23] compared the most popular reading comprehension measures used in the United States: 1/the Qualitative Reading Inventory (QRI) in which the child has to read aloud expository and narrative texts (of long length), and comprehension is assessed by retelling and short-answer comprehension questions; 2/the Gray Oral Reading Test (GORT) in which the child has to read aloud narrative passages (of medium length) and, for each passage, to answer to multiple-choice comprehension questions read by the examiner; 3/the Woodcock-Johnson Passage Comprehension (WJPC) subtest [14] [18] in which the child has to silently read short passages in which one word has been omitted, and comprehension is assessed by providing the missing word or by answering a short content question; 4/the Reading Comprehension test from the Peabody Individual Achievement Test (PIAT) in which the child must select from among four pictures the one that best represents the meaning of the short utterance s/he has read silently. Correlations between these tests were modest: they ranged from .31 between the QRI retelling and the GORT to .54 between the GORT and the WJPC. The only high correlation (.70) was between the two tests involving the reading of short passages (WJPC and PIAT). Regression analyses showed that decoding accounted for most of the variance in both the WJPC and the PIAT whereas listening comprehension accounted for most of the variance in the GORT and both QRI measures. For these reasons, reading comprehension was measured using two tasks in our study: one task used short utterances and the child had to select from among four pictures the one that best represented the meaning of the utterance s/he had read (as in the PIAT) and a second task used a narrative text of medium length, in which, after reading the text, the child had to answer questions, some of which were multiple choice (as in the GORT).

Finally, regarding the effect of the children’s SES, delays in reading and pre-reading skills have often been reported in children from low SES families [3]–[4], [24] and linguistic abilities are strongly correlated with SES [25]–[26]. Scores of low SES children, even those in the normal-readers group [27]–[30], are low, both for spoken and written language. A number of factors have been identified to explain these results: properties of mothers’ speech when talking to children [31]–[32], differences in home environment [33], and low frequency of shared reading activities at home [34]–[35]. It has been observed that children’s home literacy environment had an effect on their vocabulary in first grade and on reading comprehension in first and second grades [36]. It is therefore crucial to explore the relations between the predictors of reading comprehension in this specific population.

Despite a large body of sociological and psychological literature confirming the significant role of SES factors on reading achievement, there are very few studies on reading comprehension in children from low SES families, especially in languages with a less deep orthography than English. Recently, the predictors of reading comprehension were examined in a cohort of 181 French second graders from low SES families [2]. For these children, who achieved low scores in reading comprehension, the strongest predictors of reading comprehension were IQ, vocabulary and attention. Unfortunately, the authors did not take into account decoding level as a predictor of reading comprehension.

To our knowledge, no study has been so far performed on a large cohort of first graders from low SES backgrounds in languages with a less deep orthography than English, such as French. As indicated by descriptive and statistical data [7] [37]–[38], French orthography is quite transparent and cross-linguistics studies [6] have shown that French first graders, like Spanish first graders, obtained higher accuracy scores than same chronological age second graders from Scotland even for short (2 to 4 letters) pseudowords [6]. It is, therefore, crucial to assess the weight of the predictors of reading comprehension in French children as early as possible in their educational curriculum. Assessment of the very early predictors of reading comprehension may allow early identification of children at risk of reading difficulties, and enable early remedial training, which has been shown to be the most effective, to be set up [1].

The purpose of the present research was therefore to investigate the relations between reading comprehension, assessed at the earliest possible point in time (end of the first grade) with two tasks (one with short utterances and one with a narrative text of medium length), and the predictors of that skill (decoding, listening comprehension, and vocabulary) in a large cohort of French children from low SES families. As some researchers have indicated [39] it is difficult to establish the causal influence of SES on the development of reading skills, but one solution is to describe how it operates on reading development through the evolution of the cognitive mechanisms supporting that skill. Because the weight of the predictors of reading comprehension seems to vary across time, we investigated the contribution of the three main predictors of reading comprehension both in a 7-month longitudinal framework (from beginning to end of the first grade), and at the same point (end of the first grade).

## Method

The present study was conducted in accordance with the Declaration of Helsinki, with the written consent of each child’s parent and it was approved by the local ethics committee of the LPNC (Laboratory of Psychology and Neurocognition, CNRS, University of Grenoble) and in accordance with the ethics convention between the academic organization (LPNC-CNRS) and educational organizations.

### 1. Participants

394 French children (213 girls and 181 boys) with a mean age of 6 years 3 months (5 years 10 months to 6 years 9 month at the start of the school year), took part in this study. These children attended 30 different elementary school classes. They were all schooled in a ‘Priority Education Area’, defined as a low socio-economic catchment area, with low SES families who can be described, according to Government criteria, as families where unemployment is high or for whom low income is the predominant situation.

Nonverbal IQ scores of these children (assessed using the Progressive Matrices Standard 47 [40]: 36 problems were presented to the children, each requiring the finding of the missing part of a design from among the six options proposed), were within the norms (Table 1). In sharp contrast, at the end of the school year, 30% of our sample was more than 1 SD below the norms for reading level assessed via a French test frequently used to diagnose dyslexia labelled ‘Alouette test’ [41]. In this test, the score is based on fluency: number of words correctly read in 3 minutes. This test is designed to avoid the use of contextual information, known to be used as a compensatory strategy by poor readers and dyslexics [42]–[43]. Indeed, it includes rare words and a great deal of misleading contextual information such as ‘poison’ [poison, /z/] rather than ‘poisson’ [fish, /s/] after ‘lac’ [lake]). This test is thus assumed to assess decoding skills. Due to the reluctance of people from educational organizations to use a test known to diagnose dyslexia, only 346 out of 394 children could be assessed with this test. Therefore, their results were not taken into account for the other steps of the study. However, there were no significant differences between these 346 children and the 48 remaining children for decoding fluency assessed by pseudoword reading at the end of P1, for nonverbal IQ assessed at the beginning of P1, and for chronological age, all t<1).

**Table 1:** Summary of descriptive statistics for each of the measures as function of period.

Tests	Period 1		Period 2		
	*Mean (SD)*	*Range*	*Mean (SD)*	*Range*	*Normative data*
Nonverbal IQ (score/36)	20.79 (4.58)	14–34			20.99 (5.01) [40]
Vocabulary (score/60)	37.63 (7.10)	14–55	40.23 (7.42)	16–56	P1: 50.54 (5.49) [44]P2: 53.62 (4.75) [44]
Listening Comprehension(Utterances): %	80.26 (15.57)	24–100	85.48 (13.28)	28–100	P1: 89.42 [45]P2: 87.66 [45]
Decoding Skills: Fluency(Pseudoword-Min)	6.86 (8.42)	0–67	28.17 (11.79)	2–90	
Decoding Skills: Accuracy(Word-Pseudoword/20)	09.48 (05.42)	0–20			
Reading Comprehension (Utterances): %			68.93 (20.17)	0–100	82.7 [45]
Reading Comprehension (Text): %			52.67 (24.48)	0–100	

### 2. Procedure and Assessments

In Period 1 (P1, November) and 2 (P2, June), each child was administered tests assessing vocabulary, listening comprehension and decoding skills. Nonverbal IQ was only assessed in P1 and reading comprehension only in P2. The tasks used in both periods were the same (for vocabulary) or almost the same (for decoding skills and listening comprehension). All tasks were administered by psychologists who were trained and periodically supervised on site. In each period, assessments took place in a quiet room in the schools and lasted approximately between 45 minutes (P1) and 60 minutes (P2) per child. The children were tested individually in each period, except for the assessment of nonverbal IQ in P1, which was performed with small groups of 4–6 children, depending on the size of class). All tasks were administered the same day for all children of the same class in each period (except for nonverbal IQ). The task order for the individual assessments was chosen to maintain the attention and the interest of children and similar in P1 and P2 for all the children enrolled in the study. For both periods, the 3 first assessments were those of listening comprehension, decoding skills, and vocabulary. In P2, the assessment of reading comprehension (that included two tests) was the second to last assessment (the last one being the Alouette test) and the time lag between the listening and the reading comprehension assessment was about 40 minutes.

#### Oral language skills

Vocabulary knowledge was assessed by a standardized receptive vocabulary (TVAP) test [44] in which the children had to choose the picture (out of six) that exactly depicted the word read by the examiner. The test consisted of 30 items, each scored on a scale from 0 to 2 (2 points were awarded for the choice of the correct response and 1 point for the approximate response, e.g. the picture of ‘big house’ for the item ‘castle’). The maximum possible score was therefore 60.

The listening comprehension task, which assessed comprehension at a syntactic-semantic level, contained 25 utterances extracted from the ‘ECoSSe’” (Epreuve de Compréhension Syntaxico-Sémantique) [45] in P1 and 14 in P2 (length: 5 to 9 words). Six structures were used: active sentences; pronouns; double negation; spatial terms; relatives; passives (see Supporting information file - S1). The examiner first presented each short utterance orally. The child was then shown a page with four pictured choices, and his/her task was to select the picture which matched the utterance s/he had heard, without the possibility of hearing it again. Percentages of correct responses were calculated because maximum scores were not the same in P1 (25 points) and P2 (14 points).

#### Reading comprehension skills

As in the listening comprehension task, the task assessing reading comprehension at P2 was composed of 14 utterances (length: 5 to 9 words) from the ECoSSe [45], and the same six structures were used, but with a different wording (see (see Supporting information file - S1). The procedure was the same as the one used in the listening task, except that the child had to read each utterance aloud. He/she was then shown a page with four pictured choices and his/her task consisted of choosing the picture which matched the utterance he/she had read, without the possibility of re-reading it. This test [45] was developed to be used since Grade 1, based on the fact that, starting from this grade, a high percentage of children are able to provide correct answers to each of the test items [45]. The reading comprehension scale had 14 points. Percentages of correct responses were calculated.

Text comprehension was also assessed using an experimental task (see Supporting information file - S2). The children read a 64-words narrative text aloud, and then answered orally eight questions asked by the experimenter: two questions involving the recall of pieces of information explicitly provided in the text, four involving an inferential process, and two assessing vocabulary. The reading comprehension scale had 8 points. Percentage of correct responses was calculated.

#### Decoding skills

Two tasks were designed to assess decoding skills. One task, only used in test period 1, took into account accuracy for 10 words and 10 pseudowords (made-up of 1 to 3 letters). The scores for words and pseudowords were collapsed because they were strongly correlated (.81), with no superiority of words compared to pseudowords (means = 4.73 and 4.75, SDs = 2.84 and 3.05), as in a previous study assessing French children in January of the first grade [46]. The items used in that study were longer than those used in the present study with low SES French children and an earlier first test session (November versus January): 4 to 7 letters for words and 5 to 6 letters for pseudowords versus 1 to 3 letters for words and pseudowords in the present study. The reliability of this test was .92 (Cronbach’s alphafor the 20 items).

For both test sessions, fluency in pseudoword reading was also reported. Fluency is a composite measure which takes into account accuracy and time: very often (as in the present study) number of items correctly read in one minute. We chose pseudowords because reading this type of items is a much purer measure of decoding than reading real words (pseudowords cannot be read by sight), and, in French, at the end of the first grade (but not at the beginning) significant differences have been observed between word and pseudoword reading [46]. The choice to report fluency scores both at the beginning and end of the first grade was made to enable us to run a part of the statistics on the same measure for the two test periods. There were 30 pseudowords made-up of 1 to 5 letters in P1 and 60 pseudowords made-up of 1 to 7 letters in P2. Interestingly, the correlation between the two tasks assessing decoding skills was high in period 1 (.75). In period 2, the correlation between the two tests assessing reading fluency (pseudoword fluency and fluency in the Alouette) was very high (.88).

## Results

### 1. Descriptive Statistics

Table 1 shows the results. Our sample was comparable to norms for nonverbal IQ [40]: t(393) = −0.88; p = .37. However, for the three other tasks for which norms were available (vocabulary [44], listening and reading comprehension of utterances [45]), the means in our sample were lower than the norms (vocabulary in P1 and P2: t(393) = −44.72 and −35.92, both ps <.01; listening comprehension in P1 and P2: t(393) = −11.67 and −3.26, both ps <.01; reading comprehension in P2: t(393) = −13.03, p<.01). Children's performance improved in all tasks between P1 and P2 (for vocabulary: t(393) = +5.03; p<.01; for listening comprehension: t(393) = +5.06; p<.01; for pseudoword reading fluency: t(393) = +29.16; p<.01).

In both tasks assessing decoding skills, no floor effect appeared at any point in time. Indeed, in the test containing 20 items made-up of 1 to 3 letters, the percentage of children reading less than 10%, 25% and 50% of items were 2.2%, 23.0% and 56.8% respectively. In the test of pseudoword fluency these percentages were 30.3%, 69.5% and 88.9% respectively. In P2, these children read most of the 93 words of the reading comprehension task with short utterances (M = 85.77; SD = 13.80), and only 2% of them read correctly less than 50% of these words. Similar performances were observed for the 64 words included in the test with a story (Mean number of words correctly read in one minute = 48.25, SD = 25.2), and 26.1% of the children in the present sample read less than 50% of these words in one minute.

### 2. Correlations

The tasks used in the present study were created with different numbers of items (across time and skills) and did not have directly comparable scales. For this reason, observed scores for the variables of interest were normalized (M = 0, SD = 1). The correlation between the variables of interest in P1 and P2 (Table 2) were all significant at p<.01 (after Bonferoni correction), except those between fluency for decoding skills in P1 and vocabulary in P2 (.16), and between vocabulary in P1 and fluency for decoding skills in P2 (.10). The correlation between the two reading comprehension tasks (the task with utterances and the task with a narrative text) was high (.57). The correlations between the variables of interest in P1 and the two reading comprehension tasks in P2 were between .47 and .26: the highest being with listening comprehension (.44 for the task with utterances and .41 for the task with a narrative text) and accuracy for decoding skills (.47 and .36, respectively); the lowest being with fluency for decoding skills (.33 and .29, respectively). In P2, correlations between the variables of interest and the two reading comprehension tasks were between.60 and .38: .43 and.38 with listening comprehension, .60 and .43 with decoding skills, and .38 and .39 with vocabulary (for the first and second reading comprehension tasks respectively).

**Table 2:** Correlation matrix between all measures.

	2	3	4	5	6	7	8	9	10
1-Non verbal IQ P1	0.26**	0.31**	0.32**	0.20**	0.26**	0.21**	0.20**	0.26**	0.29**
2-Listening Comprehension (Utterances) P1		0.24**	0.37**	0.57**	0.61**	0.23**	0.50**	0.44**	0.41**
3-Decoding skills (Fluency) P1			0.71**	0.19**	0.17**	0.47**	0.16	0.33**	0.29**
4-Decoding skills (Accuracy) P1				0.28**	0.26**	0.53**	0.30**	0.47**	0.36**
5-Vocabulary P1					0.51**	0.10	0.61**	0.33**	0.36**
6-Listening Comprehension (Utterances) P2						0.18**	0.45**	0.43**	0.38**
7-Decoding skills (Fluency) P2							0.18**	0.60**	0.43**
8-Vocabulary P2								0.38**	0.39**
9-Reading Comprehension (Utterances) P2									0.57**
10-Reading Comprehension (Text) P2									

** p<.01 after Bonferroni correction.

### 3. Regression Analyses

Regression analyses were performed in order to find which of the P1 and P2 variables best predict reading comprehension in P2. They were computed with the two reading comprehension tasks (with short utterances and with a narrative text) as the dependent variable and variables of interest in P1 and P2 as predictors.

The analysis reported in Table 3 showed that the variables of interest in P1 accounted for 31% of the variance in the reading comprehension task using short utterances in P2. Only listening comprehension and decoding skills (accuracy) made a significant contribution to the prediction. The percentage of explained variance was lower when fluency (instead of accuracy) was taken into account (26%, see Table 4). As in the previous analyses, only listening comprehension and decoding skills (fluency) made a significant contribution to the prediction. The variables of interest in P2 accounted for 49% of the variance in reading comprehension (Table 4). The contributions of the 3 variables of interest were significant, with decoding skills (fluency) proving to be the best predictor.

**Table 3:** Standard multiple regression analyses with the reading comprehension of utterances as dependent variable and variables of interest at P1 as predictors (for decoding skills: accuracy scores for word and pseudoword reading).

R^2^	Predictor variables	*β*	Proportion of variance accounted by	*P*
P1–P2: 31%	Listening Comprehension P1	.26	5.48%	<.01
	Decoding skills P1	.33	11.15%	<.01
	Vocabulary P1	.07	0.52%	.15
	Nonverbal IQ P1	.07	0.63%	.12

**Table 4:** Standard multiple regression analyses with the reading comprehension of utterances as dependent variable and variables of interest at P1 and P2 as predictors (for decoding skills: fluency in pseudoword reading).

R^2^	Predictor variables	*β*	Proportion of variance accounted by	*P*
P1–P2: 26%	Listening Comprehension P1	.31	7.67%	<.01
	Decoding skills P1	.21	5.15%	<.01
	Vocabulary P1	.09	0.73%	.09
	Nonverbal IQ P1	.09	0.94%	.06
P2–P2: 49%	Listening Comprehension P2	.25	8.89%	<.01
	Decoding skills P2	.52	33.99%	<.01
	Vocabulary P2	.18	4.52%	<.01

The analysis reported in Table 5 showed that the variables of interest in P1 accounted for 25% of the variance in the reading comprehension task with a narrative text in P2. Unlike the previous analysis, all the predictors (listening comprehension, accuracy for decoding skills, vocabulary and nonverbal IQ) significantly contributed to the prediction. The results were very similar when fluency for decoding skills was taken into account (see Table 6). The variables of interest in P2 accounted for 32% of the variance in reading comprehension (Table 6). As in the analysis of the reading comprehension task using short utterances, the contributions of the 3 variables of interest were significant, with decoding skills (fluency) proving to be the best predictor.

**Table 5:** Standard multiple regression analyses with the reading comprehension of a story as dependent variable and variables of interest in P1 as predictors (for decoding skills: accuracy scores for word and pseudoword reading).

R^2^	Predictor variables	*β*	Proportion of variance accounted by	*p*
P1–P2: 25%	Listening Comprehension P1	.21	3.52%	<.01
	Decoding skills P1	.20	4.20%	<.01
	Vocabulary P1	.15	2.04%	<.01
	Nonverbal IQ P1	.14	2.13%	<.01

**Table 6:** Standard multiple regression analyses with the reading comprehension of a story as dependent variable and variables of interest in P1 and P2 as predictors (for decoding skills: fluency for pseudoword reading).

R^2^	Predictor variables	*β*	Proportion of variance accounted by	*p*
P1–P2: 24%	Listening Comprehension P1	.24	4.63%	<.01
	Decoding skills P1	.16	2.89%	<.01
	Vocabulary P1	.16	2.24%	<.01
	Nonverbal IQ	.14	2.19%	<.01
P2–P2: 32%	Listening Comprehension P2	.20	4.64%	<.01
	Decoding skills P2	.34	14.93%	<.01
	Vocabulary P2	.24	6.09%	<.01

## Discussion

The contribution of the main predictors of reading comprehension (listening comprehension, vocabulary and decoding) was examined in 394 French first graders from low SES families. Our first important finding was that, as observed in studies on English children [1], [3], or French children [4], the low SES children in this study showed impairments in their spoken and reading skills. Indeed, their scores were below the norms for decoding skills assessed with the Alouette at the end of first grade, and for vocabulary and listening comprehension both at the beginning and end of the same grade. Otherwise, our sample was comparable to the norms for nonverbal IQ, as observed for children from low SES levels in previous studies [24].

Regarding the predictors of reading comprehension, the contribution of listening comprehension to the understanding of either written utterances or a narrative text was equivalent in P1 and P2. However, the involvement of listening comprehension was lower in the task using a narrative text than in the task using short utterances, a result that could be due to the fact that the items used in the latter task were very similar to those used in the listening comprehension task, but not to those used in the task with a narrative text.

In contrast, the contribution of decoding to reading comprehension highly increased between P1 and P2, irrespective of the reading comprehension task used. The results also indicated that the predictive power of very early decoding skills (in P1) was greater when these skills were assessed with accuracy scores for short items (1 to 3 letter words and pseudowords) than with fluency for a list of pseudowords including longer items. In addition, these results indicated that the relative weight of decoding to reading comprehension was greater in the reading comprehension task using short utterances than in the one using a narrative text.

These results are not consistent with those observed in a previous study on French first graders [12] in which, from the end of the first grade, the contribution of listening comprehension to the prediction of reading comprehension was greater than that of decoding skills, whereas the opposite was observed in studies on English children [e.g. 14]. The difference in the relative weight of decoding versus listening comprehension on reading comprehension is generally attributed to orthographic transparency. Inconsistency is assumed to slow down reading development [7]; and the effect of orthographic consistency on such development can explain the greater contribution of decoding to reading comprehension in languages with a deep orthography, such as English [14]. The fact that the sample of this study showed impairment in decoding skills, as did children from low SES families evaluated in previous studies [2]–[4] [26], could explain why decoding was the most important predictor in that study despite the relative transparency of French orthography [37]–[38]. The French results, suggesting that listening comprehension had greater contribution to reading comprehension than decoding skills at the end of the first grade [12], could be explained by two factors that were not taken into account in that study: the children’s vocabulary level, and their high socio-economic status (the children enrolled in that study were from areas of Paris which are rather socially privileged). We can therefore assume that their environment allowed at least most of them to master decoding skills quickly and efficiently,; this could have led to an increase in the weight of listening comprehension to the detriment of decoding skills in that study [12]. Further studies are needed to better understand the relative weight of decoding versus listening comprehension on reading comprehension in the initial stages of reading acquisition in different populations (low vs. high SES), different orthographies (shallow vs. deep), and taking into account the children’s vocabulary level.

In the present study, oral vocabulary was assessed with a receptive test, as in other studies with first graders [13]–[14] [17]. A first result is that the impact of vocabulary was lower on the task that involved short utterances than on the one with a story. These results can be explained by the fact that the words used in the first case are frequent [45], whereas there are some words of a lower level of frequency in the task with a story (‘la mare’ [the pond], ‘se précipiter’ [to rush], ‘secours’ [rescue], ‘tronc d’arbre’ [tree trunk], ‘flotter’ [to float], ‘sauver’ [to save], ‘grimper’ [to climb], ‘le rivage’ [the shore]). The results also indicate that the weight of vocabulary on reading comprehension increased from the beginning to the end of the first grade. This can be due to the fact that vocabulary is assumed to be connected to orthographic knowledge [14], a knowledge that increases sharply during the first years of schooling (e.g. in Dutch [17], in French [46], in English [13] and for a review, see [7]). The significant weight of vocabulary in predicting reading comprehension as early as first grade is consistent with some results found in Dutch [17] and in English [13], but it contradicts other results observed in English [14]. This difference could be due to the fact that, in that study with English first graders [14], in addition to pseudowords, irregular word reading was entered in the regression model. The correlation between irregular word reading and reading comprehension was high (.77), as was the correlation between pseudoword reading and reading comprehension (.79). However, the correlation between irregular word reading and vocabulary was weaker, but significant (.38). As a result, the significant part of the variance in reading comprehension explained by irregular word reading may have hidden the part of the variance in that skill that could have been be explained by vocabulary. Further studies are needed to better understand these results.

The use of two reading comprehension tasks in the present study allowed us to show that nonverbal IQ made a significant contribution to the prediction of reading comprehension only when assessed using a narrative text. The contribution of decoding to the prediction was, on the other hand, greater when reading comprehension was assessed using short utterances. These results are in line with those found in a study with English students [23] and suggest that the two reading comprehension tasks we used do not measure exactly the same components of reading comprehension. In the reading comprehension task using short utterances (length: 5 to 9 words) decoding difficulties may be more detrimental than in the other task, involving a 64 word narrative text. In a short utterance there often are no other words that the child could use to determine the meaning of the words s/he is not able to decode whereas, in a longer passage, the use of the context (for instance description of events) can help to guess some of these words. The finding that decoding accounted for much less of the variance in the reading comprehension of a medium length narrative text, compared to short utterances, corroborates that hypothesis. The length of the passages in the reading comprehension of a narrative text provides the contextual support needed to rectify at least some decoding problems; and it is well known that words in context are easier to read than isolated words especially for poor and very poor readers [42]–[43]. In addition, some researchers have suggested that this kind of task (narrative text with multiple-choice comprehension questions) assesses not only reading comprehension, but also reasoning based on prior knowledge [47]. Our findings are partially consistent with this hypothesis because nonverbal IQ (reasoning test) accounted for an additional part of the variance in the reading comprehension of a narrative text, but not in the other reading comprehension task. Despite these differences, the correlation between the two reading comprehension tasks was quite high (.57), and higher than the one observed in a study with English students [23] between two tasks similar to those used in the present study, the PIAT and the GORT (.51).

## Conclusion

Overall, the results of the present study strongly highlight the view that comprehension is a complex cognitive construct, and successful reading comprehension is the result of a confluence of skills: decoding and listening comprehension, as predicted by the simple view of reading [5], plus (but with a lesser weight and a lesser consistency) oral vocabulary. The present results also indicate that the relationship between these predictors and reading comprehension could vary as a function of the specific task used to assess comprehension. However, because variance large part of variance remains unexplained, further research should also consider the relevance of other potentially important predictors of reading comprehension: especially, working memory [48] and attention [2].

Otherwise, the results of the present study bear practical implications for at risk populations. The risk of a child developing reading comprehension difficulties is smallest when s/he makes age-appropriate progress in each component skill. More education and training help each child move forward in each skill, and ensure against failure. Given the associations and mutual influence between, decoding, vocabulary skills, listening comprehension and reading comprehension, all of these abilities should be emphasized during children’s reading education. More importantly, while studies in transparent orthographies emphasize the importance of listening comprehension as early as the first grade, our results showed that in low SES French children, decoding was the most important predictor of reading comprehension at the end of the first grade.

Finally, in international assessments (PIRLS [49] and PISA [50]) reading comprehension was evaluated without considering the level of decoding, listening comprehension and vocabulary. This type of evaluation does not allow us to clearly establish, for ‘children with difficulties’, what exactly may explain their difficulties, and therefore provide the support they need. Early interventions are known to be the most effective [1]. However, PIRLS assessments are intended for children aged 9–10 and those of PISA for students aged 15 years. This is too late. The present study shows that it is possible to assess two aspects of reading (decoding skills and reading comprehension) very early on, even in low SES children by using tests appropriate for this population. It is our hope that the use of an assessment such as the one designed for the present study could inspire educational practices for children with specific needs in the domains of written and spoken language.

## Supporting Information

Figure S1 **Listening and reading comprehension:** Structures tested and examples. The mean % (and ranges) of children providing a correct response for the different utterances are those obtained by 1^st^ graders (chronological age: 84 to 95 months) in the ECoSSe [45] for the reading comprehension task.
(DOCX)

Figure S2 **Reading comprehension: Story.**
(DOCX)
