# Supplementary material for: Reading Comprehension in a Large Cohort of French First Graders from Low Socio-Economic Status Families: A 7-Month Longitudinal Study
Source: PLoS One. 2013 Nov 8;8(11):e78608. doi: 10.1371/journal.pone.0078608 (PMC3826761; doi:10.1371/journal.pone.0078608)
Supplement: Figure S1 — Listening and reading comprehension: Structures tested and examples. The mean % (and ranges) of children providing a correct response for the different utterances are those obtained by 1st graders (chronological age: 84 to 95 months) in the ECoSSe [45] for the reading comprehension task. (DOCX) [file pone.0078608.s001.docx]

**SUPPORTING INFORMATION FILE – Figure S1**

| **Structures** | **Examples of utterances used for the assessment of listening (LC) and reading (RC) comprehension** | **Examples of picture**  **(LC or RC)** |
| --- | --- | --- |
| **Active sentences** | **LC**- La fille pousse le cheval.  [The girl pushes the horse]  **RC**- La dame pousse la vache.  [The woman pushes the cow]  Mean % (RC [45]): 93.30 (Range: 91.75 and 94.84) | **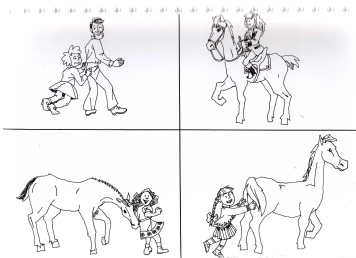LC** |
| **Passives**  **sentences** | **LC**- La fille est poursuivie par le cheval.  [The girl is chased by the horse]  **RC**- Le garçon est poursuivi par le mouton.  [The boy is chased by the sheep]  Mean % (RC [45]): 78.35 (Range: 72.16 and 84.54) | **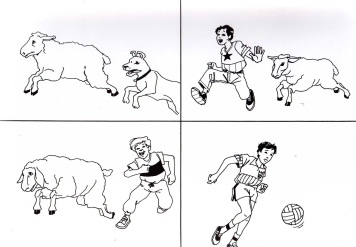RC** |
| **Pronouns** | **LC-** L’éléphant les porte.  [The elephant carries them]  **RC-** La vache les regarde.  [The cow watches them]  Mean % (RC [45]): 92.78 (Range: 91.75 and 93.81) | **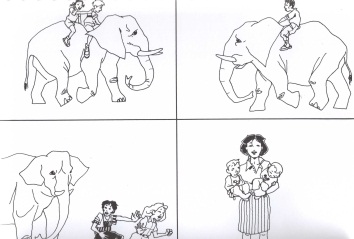LC** |
| **Double negation** | **LC-** Ni le garçon ni le cheval ne courent.  [Neither the boy nor the horse run]  **RC-** Le garçon n’a ni chapeau ni chaussure.  [The boy has no hat and no shoes]  Mean % (RC [45]): 95.35 (Range: 90.72 and 99.97) | **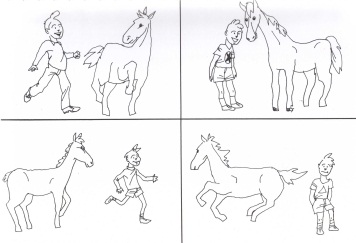LC** |
| **Spatial terms** | **LC-** Le crayon est derrière la boîte.  [The pencil is behind the box]  **RC-** La boîte est derrière la tasse.  [The box is behind the cup]  Mean % (RC [45]): 78.09 (Range: 71.13 to 89.69) | **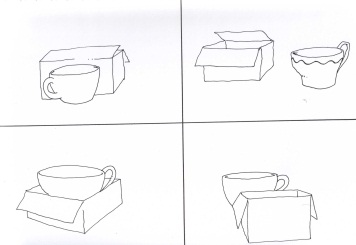RC** |
| **Relatives** | **LC-** Le crayon qui est sur le livre est jaune.  [The pencil which is on the pencil is yellow]  **RC-** L’étoile qui est dans le cercle est rouge.  [The star which is in the circle is red]  Mean % (RC [45]): 73.21 (Range: 72.16 to 75.26) | **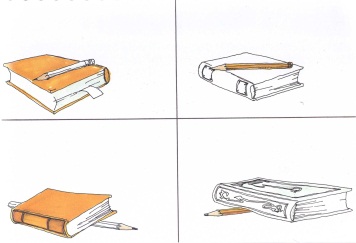LC** |
