# Supplementary material for: Reading Comprehension in a Large Cohort of French First Graders from Low Socio-Economic Status Families: A 7-Month Longitudinal Study
Source: PLoS One. 2013 Nov 8;8(11):e78608. doi: 10.1371/journal.pone.0078608 (PMC3826761; doi:10.1371/journal.pone.0078608)
Supplement: Figure S2 — Reading comprehension: Story. (DOCX) [file pone.0078608.s002.docx]

**SUPPORTING FILE INFORMATION – Figure S2**

La petite poule blanche est tombée dans la mare.

[The little white hen fell into the pond].

La grande poule noire se précipite à son secours mais elle tombe elle aussi dans la mare et se demande "Que faire? Que faire?".

[The big black hen rushes to his aid but also falls into the pond and wondered "What to do What to do." ].

L’autre poule dit "Regarde ce tronc d’arbre qui flotte, il peut nous sauver".

[The other hen said "Look what tree trunk floating, it can save us."].

Elles grimpent sur le tronc d’arbre et crient "Ouf, nous allons pouvoir regagner le rivage!"

[They climb on the tree trunk and shout "Phew, we can reach the shore!"]

**Questions:**

1) Example of question involving the recall of pieces of information explicitly provided in the text:

-What is the color of the little hen? (white).

2) Example of question involving an inferential process:

-What important object saw the little hen? (a tree trunk).

3) Example of question about vocabulary:

-In "The big hen rushes [se précipite]…"what is the meaning of "rush"? Go slowly? Fall over a precipice? Go quickly?
